# Spread and Scale-Up of a Region-Wide Telehealth Navigation Service in a Rural Context: Qualitative Process Evaluation

**DOI:** 10.2196/64734

**Published:** 2025-06-23

**Authors:** Mary Malakellis, Anna Wong Shee, Laura Alston, Vincent L Versace, Pheona Griffith, Jade Odgers, Kevin Mc Namara

**Affiliations:** 1 Deakin Rural Health School of Medicine Deakin University Warrnambool Australia; 2 Grampians Health Ballarat Australia; 3 Colac Area Health Colac Australia

**Keywords:** telehealth, rural, implementation, health services, frequent presenters, case management

## Abstract

**Background:**

Few evaluations of interventions adapted or developed for a rural context have been conducted, and little is known about how to spread and scale up interventions from a metropolitan to a rural context. Similarly, evidence on implementation processes for interventions to address people with frequent potentially avoidable presentations (hereafter referred to as frequent presenters) to the emergency department is scarce in rural settings. In this study, Patient Watch, a telehealth case management program modeled on a metropolitan service, was implemented in a rural context to support frequent presenters.

**Objective:**

This study aims to understand the spread, scale-up, and sustainability of Patient Watch in a rural context to inform the potential for transferability to other contexts.

**Methods:**

The study design was a qualitative process evaluation, collecting and synthesizing data obtained from semistructured interviews (n=10) with stakeholders, including executives, managers, physicians, and frontline staff; document review, including steering committee meeting minutes (n=17) and other relevant documents (eg, planning documents, workflow plans, consumer information, and standard operating procedures; n=20); observation of steering committee meetings of 1-hour duration (n=11); and 1 on-site visit with the Patient Watch team at the lead agency. The data were categorized into themes using thematic analysis.

**Results:**

Three themes were constructed from the data: (1) health care system complexity, (2) context drives adaptation and innovation, and (3) autonomy. Preexisting health system factors, including financial resources, workforce constraints, and infrastructure challenges, influenced organizational capacity to scale-up Patient Watch. Continuous adaptation in response to health system factors was essential for tailoring Patient Watch. Local autonomy facilitated adaptation to address variations across local contexts; however, restrictive governance impeded the ability to respond to local challenges.

**Conclusions:**

Our findings emphasized how rural contextual factors influenced the spread, scale-up, and future sustainability of Patient Watch and provided a greater understanding of implementing and adapting a metropolitan model in a rural context. Future research should acknowledge health system complexity; prioritize ongoing adaptations in various contexts; and integrate a balance of distributed leadership to improve the spread, scale-up, and sustainability of telehealth case management interventions.

## Introduction

### Background

Emergency department (ED) overcrowding is a global public health concern [1]. Individuals who have frequent potentially avoidable ED presentations (hereafter referred to as frequent presenters) represent a disproportionately high number of ED presentations [2]. Frequent presenters, variably defined as presenting between ≥3 and ≥7 times per year, are heavy users of the broader health care system and frequently have complex health needs with a mix of physical, mental, and social issues [3].

People living in rural areas are more likely to present to the ED [4], and rural EDs have comparable rates of frequent potentially avoidable presentations to metropolitan EDs [5,6]. Furthermore, people living in rural areas experience higher burdens of disease, chronic conditions, and health-risk factors [7,8], compounded by limited access to health care services, higher out-of-pocket costs, and health workforce shortages [9], which are longstanding challenges in rural areas. A recent study confirmed that the relevant health care priorities to address in rural areas included access issues, challenges navigating the health care system, the lack of health care professionals, travel costs, and poor internet coverage [10]. The causes and drivers of frequent presentations to rural EDs are complex and involve interrelated factors, including a lack of health service access, poor care coordination, patient needs, and a lack of knowledge and skills [11]. Notably, the systems approach used in this study [11] has not been applied in other contexts, including a metropolitan context. However, other studies have identified individual factors, including low socioeconomic status, high levels of health care use (other than the ED), and multiple chronic conditions, in a metropolitan context, to predict frequent presentations to the ED [3,12]. Similarly, qualitative analysis of patient perspectives suggests that negative experiences with the health care system, challenges associated with low socioeconomic status, and significant mental and physical chronic disease burden are associated with frequent presentations [13].

Case management, a collaborative approach to coordinate and integrate care to meet patient needs [14], has been frequently implemented to improve care for frequent presenters [15-17]. Recent literature reviews report positive outcomes, including reductions in ED presentations and costs with face-to-face case management [15-17] and telehealth case management [18]. More broadly, telehealth case management models in rural populations have been associated with benefits to patients (eg, reduced costs, travel time, and improved access to care) and health care providers (eg, reduced staffing costs and lower on-site health care use), suggesting that these models are acceptable and feasible [19]. However, evidence relating to intervention fidelity, implementation processes [20,21], spread, scale-up, and sustainability remains scant for face-to-face and telehealth case management. Furthermore, very few interventions have been developed for a rural context [22]. A recent systematic review of interventions globally found that a few studies had specifically adapted interventions in a rural context [23]. Similarly, there is limited evidence of interventions for frequent presenters designed or adapted specifically for a rural context. This represents a considerable evidence gap for a vulnerable population with low levels of health service access.

The spread, scale-up, and sustainability of successful interventions in new settings are challenging, with many promising interventions failing to bring about large-scale changes [24]. Operational definitions of spread, scale-up, and sustainability vary widely, and the terms are used interchangeably [25]. The definitions of spread, scale-up, and sustainability used in this study are as follows: spread refers to transfer of an intervention to other organizational settings beyond the original setting, scale-up refers to development of infrastructure to support widespread implementation across several settings to expand coverage, and sustainability refers to maintaining the intervention through adaptation to context over time [25,26]. Despite a proliferation of theories, models, and frameworks to guide implementation, relatively few have been developed for spread, scale-up, and sustainability, and those that do rely on a limited evidence base [27,28]. The dynamic sustainability framework (DSF) [29] and the nonadoption, abandonment, scale-up, spread, and sustainability (NASSS) framework [30] adopt a complex systems approach, acknowledging unpredictability and viewing interventions as part of a complex system consisting of multiple interacting components. The DSF emphasizes ongoing adaptations over time to establish an optimal but dynamic fit between the intervention, the implementation context, and the broader system [29]. NASSS seeks to understand the success of technology-supported health or social care interventions [30]. While these frameworks offer a strong approach for facilitating the spread, scale-up, and sustainability of interventions, an equity lens and a more intentional focus on the diversity of rural areas need to be considered [31].

Although there is strong interest in the spread, scale-up, and sustainability of case management in health care, there is little information available on how to do this, particularly in rural settings. Our study evaluated the spread of Patient Watch, a telehealth program using a case management model of care to manage the care of frequent presenters, from a metropolitan setting and the subsequent scale-up across a rural region in Victoria, Australia.

### Objective

This study aimed to explore how contextual factors influenced the spread, scale-up, and sustainability of Patient Watch in a rural context.

## Methods

The study design is a qualitative process evaluation.

### Ethical Considerations

Ethics approval was received from the Deakin University Human Research Ethics Committee (2022-174) and the Ballarat Health Services and St John of God Human Research Ethics Committee (HREC/78891/BHSSJOG-2022-317489(v3)). Participants were informed about the study and participation was voluntary. Written informed consent was obtained from all participants for semistructured interviews: verbal consent was obtained from the steering committee to observe meetings and from frontline staff for the on-site visit. All data were fully anonymised to ensure privacy and confidentiality. All participants were given an Aus $20 (Aus $1=US $0.66) gift voucher in recognition of the time taken to participate in the semistructured interviews.

### The Patient Watch Model

**Figure 1:**
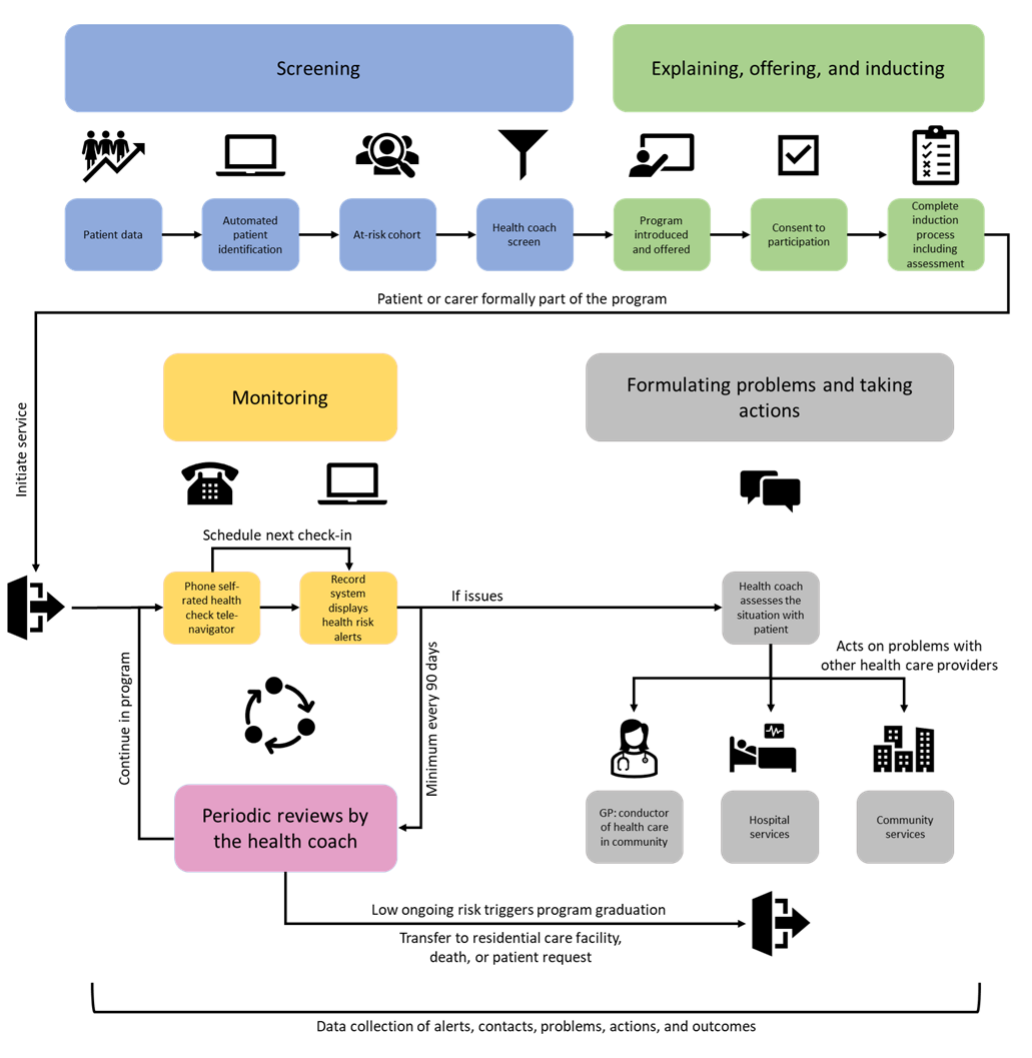
Schematic of the adapted Patient Watch program for the rural context. GP: general practitioner.

The Patient Watch model was developed for an Australian metropolitan population at Monash Health, Victoria, to reduce avoidable hospitalizations and ED presentations through monitoring patient health with a transdisciplinary team supporting care, and it showed a significant reduction in acute hospital bed days [32]. Figure 1 shows the schematic of the Patient Watch model as adapted from the metropolitan model.

Patient Watch has five core functions: (1) screening for eligibility; (2) explaining, offering, and inducting; (3) monitoring health and well-being; (4) formulating problems and taking actions; and (5) periodic reviews (K Stockman, unpublished data, April 2021). Screening identified eligible patients using a modified version of the HealthLinks Chronic Care algorithm [33] to predict likely future frequent presenters or from internal or general practitioner (GP) referral. Candidates for Patient Watch were reviewed by the health coach (registered nurse) for appropriateness (eg, candidate was excluded if they were inpatients or living in a residential care facility). A lay tele-navigator support (TNS) worker contacted eligible candidates and provided information about the program. Interested candidates were then contacted by the health coach; consent was obtained; and their current medical, emotional, and social situation was assessed via telehealth. Monitoring was conducted through conversational semistructured phone calls by TNS workers to track patients’ self-rated health and needs at regular intervals (typically weekly). TNS workers were supported by the Patient Journey Record system, providing real-time support to detect adverse changes in patient biopsychosocial trajectories [34]. Formulating problems and taking actions in response to health decline required a health coach to coordinate interventions with the community, hospital, and social service providers. Periodic reviews were conducted every 3 months by the health coach to determine if the patient’s health had sufficiently stabilized to graduate from the program or recommend further monitoring.

Key adaptations to the rural Patient Watch model included the recruitment of registered nurses as health coaches instead of health professionals from multiple disciplines providing integrated care and the use of telehealth by the health coach instead of face-to-face home visits. Home visits were offered if necessary. The reason and timing of adaptations are described in the Results section of this paper.

### Implementation Context

Implementation of Patient Watch involved a collaboration of 7 health services of varying size and remoteness across a rural region in Victoria, Australia, to address frequent presentations. There is considerable heterogeneity in the use of geographic classification defining rurality [35,36]. In this study, we used the Australian Department of Health and Aged Care’s definition of rural and the modified Monash (MM) model, which classifies metropolitan, regional, rural, and remote areas into 7 categories with rural areas defined as those including all categories (MM2-7), except the category “metropolitan areas” (MM1) [37]. This study covered areas of regional centers (MM2), large rural towns (MM3), medium rural towns (MM4), and small rural towns (MM5).

The lead agency was the main public referral health service for the region, located in the regional center, with a catchment population of >250,000 people and a region spanning 48,500 km^2^. In November 2021, a new health service was established after the amalgamation of the lead agency with 3 of the health services [38]. Amalgamation of the new health service occurred between July 2021 and December 2022 [38]. The lead agency spread Patient Watch initially, followed by scaling-up to other health services across the region. Foundation, launch, and growth of initial spread efforts at the lead agency and scale-up across the rural region coincided with the amalgamation of the new health service.

**Figure 2:**
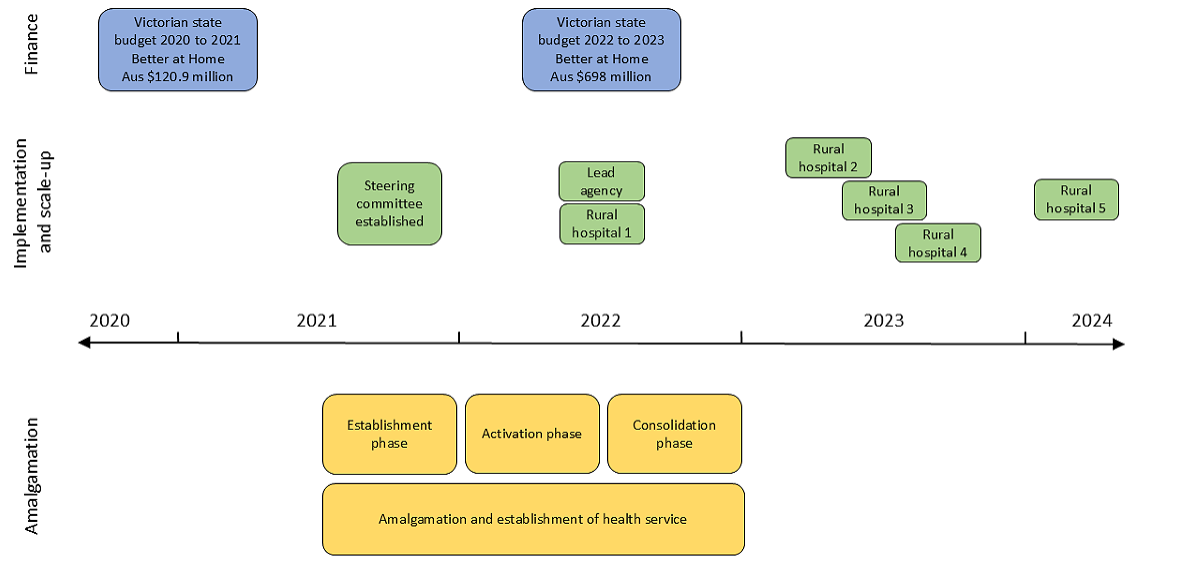
Schematic of timeline for the spread and scale-up of Patient Watch.

Funding was obtained from Better at Home (Aus $120.9 million over 3 years), a 2020 to 2021 Victorian budget initiative to deliver hospital services within patients’ homes through home-based and virtual care [39]. A further commitment to expand the program (Aus $698 million) was announced in the 2022 to 2023 Victorian budget [39]. The lead agency managed funds received with sites subcontracting to receive payment for services rendered for local Patient Watch programs, except for rural hospital 1, which applied for and maintained its own funding. Figure 2 shows a schematic of the timeline for the implementation of Patient Watch.

### Data Collection

We collected and synthesized data from the following sources.

#### Observation

Researchers (MM, KM, and AWS) attended the steering committee meetings of 1-hour duration from November 2022 to February 2024 (n=11) about Patient Watch, its operationalization, spread, and scale-up, with field notes taken by authors (MM and KM). On average, steering committee meetings were attended by 11 (SD 2.9; range 7-15) members, and they were representatives from health services across the rural region, including project leads, executive and assistant directors, clinicians, the Hospital Admissions Risk Program (HARP) manager, lead health coach, TNS workers, and researchers. One on-site visit (April 2023) with the Patient Watch frontline team located at the lead agency was conducted, involving informal conversations to gain familiarity and engage with their daily activities, with field notes taken by 1 author (MM).

#### Document Analysis

Documents were collected from May 2022 to February 2024 and included steering committee meeting minutes (n=17) and other relevant documents, including planning documents, workflow plans, consumer information, consumer feedback forms, GP information, guidelines, standard operating procedures (SOPs), checklists, and other miscellaneous documents (eg, geographic map of planned scale-up; n=20). The steering committee meeting minutes were sourced from the minute taker, and other relevant documents were either attached to the steering committee meeting minutes or sourced from the project manager, project support officer, or frontline staff at the lead agency.

#### Semistructured Interviews

Semistructured interviews (n=10) were conducted online using Microsoft Teams between May and June 2023, with interview guides informed by the Consolidated Framework for Implementation Research (Multimedia Appendix 1) [40]. Interviews were conducted during the early spread at the lead agency and rural hospital 1 and before scale-up for rural hospitals 2 to 5. Interviews lasted between 40 and 70 minutes and explored stakeholders’ perceptions of program strengths and limitations, early program implementation, planned scale-up across the region, and future sustainability.

### Included Participants

Participants included members of the Patient Watch steering committee involved in the planning, spread, and scale-up of the program. Researchers provided information about the study at a monthly meeting and were provided with the email contacts of eligible participants (all steering committee members) with permission from the project lead. Convenience sampling was used to obtain a variety of organizational and individual perspectives [41]. Snowball sampling was also used, whereby participants were asked to identify other potential participants from within their immediate team or organization [41].

In total, 22 people were invited to participate, including 15 (68%) steering committee members and 7 (32%) people identified by snowball sampling. Those invited to participate included executives (n=4, 18%), managers (n=12, 55%), frontline staff (n=4, 18%), physicians (n=1, 5%), administration staff (n=1, 5%), and they represented the lead agency (n=15, 68%), rural hospital 1 (n=1, 5%), rural hospital 2 (n=3, 14%), rural hospital 4 (n=1, 5%), and other rural hospitals where negotiation for scale-up was underway (n=2, 9%; rural hospitals 2, 3, and 5 amalgamated with the lead agency before scale-up).

All study participants received written information about the project via email, with a reminder email sent fortnightly for 6 weeks.

### Data Analysis

All interviews were conducted, audio recorded, and transcribed verbatim by the first author (MM). We used reflexive thematic analysis following the 6-phase approach by Braun and Clarke [42], informed by a critical realist approach. Critical realism acknowledges there is a reality beyond what is observable, and we can make sense of reality through our experience and interpretation [43]. Critical realism recognizes the interaction between context, causal mechanisms, and the outcomes of an intervention. This analytic lens enabled the identification of how a complex intervention, such as Patient Watch, is effective and the influence of context on implementation and scale-up rather than assuming that the program alone affects change [44].

Reflexive thematic analysis is a method for identifying, describing, analyzing, and reporting patterns within data using a theoretically flexible 6-phase approach [42]. This approach combined inductive and deductive reasoning to draw on existing theoretical frameworks, such as the Consolidated Framework for Implementation Research [40], NASS [30], and DSF [29], and offered considerable freedom for identifying new ones [42]. Data analysis was conducted by the primary author (MM), in collaboration with AWS and KM.

Interviews were transcribed immediately following each interview, reviewed independently, and discussed by MM and KM, and the findings informed subsequent interviews. Once all interviews were conducted, they were read in their entirety and then reread numerous times, and initial ideas for coding were noted. Initial analytic observations and interpretations shifted between inductive and deductive modes, focusing on the experience of spread and scale-up of Patient Watch, remaining open to all possible interpretations. Codes included details about the components of Patient Watch, positive and negative experiences of spread and scale-up, health system, health service and patient differences across the region, and sustainability. Codes were assembled into initial candidate themes, explored for distinctiveness or overlap, and reorganized to better reflect the themes. Themes were identified on a “surface” semantic and latent level, and extracts were checked for consistency within each code and theme. Reflexive thematic analysis was supported by NVivo (version 14; Lumivero).

### Reflexivity

Data collection and analysis were informed by researchers’ previous experience, knowledge gained from reviewing available literature, and experience with implementing similar programs into health services. Reflexivity was part of the approach, through collaborative discussion (with AWS and KM), independent notes taken (MM and KM), and debriefing sessions following each interview to reflect and share feedback. This ensured that interpretation of findings reflected the data and balanced with individual views.

An electronic audit trail included notes taken following each interview, and further notes taken as transcripts were read and reread. Additional material included lists of codes, tables of codes and themes, and the development of themes and thematic maps. The inclusion of different data sources allowed multiple stories and diverse experiences to be captured, increased the participation of a broader group of eligible stakeholders, and enhanced understanding and interpretation.

The team consisted of researchers with a clinical background (eg, pharmacy and physiotherapy) and with extensive research experience in health service research and public health in a rural context. MM and KM are affiliate researchers with the lead agency and did not have any previous relationship with the steering committee members. AWS is employed by the lead agency and was a member of the steering committee. Practicing reflexivity acknowledged our role in the research and the previous experiences, assumptions, and beliefs we brought to the research process. We approached the research with a diversity of perspectives (MM as a PhD candidate, KM with an academic background, and AWS from within a health service) and constructed a shared understanding of the spread and scale-up of Patient Watch, mediating any differences of opinion through considerable and continuous discussion.

## Results

### Overview

A total of 10 participants were interviewed (steering committee: n=5, 50%; external to the steering committee: n=5, 50%), and their characteristics are shown in Table 1. Most participants were female (n=7, 70%), aged 49.6 (SD 3.9) years, and with a clinical background (n=9, 90% registered nurses, allied health professionals, or physicians). Participants had 25.1 (SD 7.2) years of experience since registration; had 15.4 (SD 11.9) years of experience working in the region; and were executives, managers, physicians, and frontline staff. Limited demographic information is provided to protect participant confidentiality.

**Table 1:** Characteristics of semistructured interview participants (n=10).

Demographic characteristics		Values
Sex: female, n (%)		7 (70)
Age (y), mean (SD)		49.6 (3.9)
Health professional, n (%)		9 (90)
Experience since registration (y), mean (SD)		25.1 (7.2)
Duration of working in the region (y), mean (SD)		15.4 (11.9)
**Position, n (%)**		
	Executive	2 (20)
	Frontline staff	4 (40)
	Physician	1 (10)
	Manager	3 (30)
Steering committee members, n (%)		5 (50)
External to the steering committee, n (%)		5 (50)

### Themes

**Figure 3:**
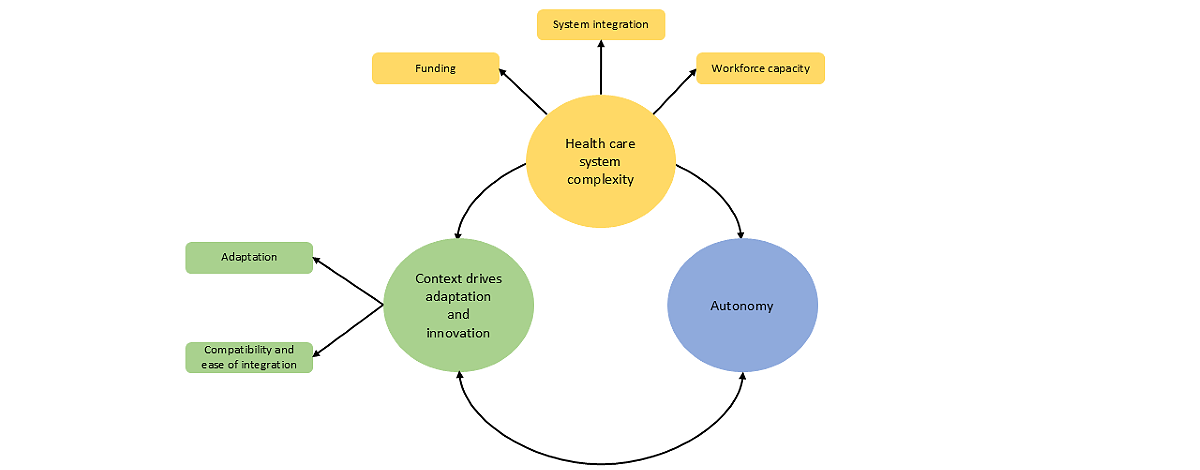
Thematic map showing 3 main themes.

Three themes were constructed from the data: (1) health care system complexity, (2) context drives adaptation and innovation, and (3) autonomy. A visual map was created, consisting of themes and subthemes, and the connections between themes were mapped (Figure 3).

#### Theme 1: Health Care System Complexity

##### Overview

This theme represented the complexity of the health care system, that is, the external factors that influenced the spread and scale-up. Participants recognized that health services operated as part of a large and complex system. Key factors contributing to this complexity were funding, system integration, and workforce capacity. These factors also had important implications for adaptations, described in theme 2, as did funding for autonomy, described in theme 3.

##### Funding

Spread and scale-up of Patient Watch was initially financed through state government funding [39]. The lead agency was allocated funding to deliver health care through home-based and virtual care across the rural region, except for rural hospital 1, which applied for and maintained its own funding. Funding provided the momentum to “get things started” (SH10; interview; lead agency), particularly given previous unsuccessful attempts to access other funding sources to support spread (eg, research grants). However, there was recognition that funding would be insufficient for the long-term sustainability of Patient Watch:

...everyone’s concern and the funding initially...didn’t really allow us to be funded until we escalated someone’s care...

SH10; lead agency; interview

...there’s no extra money in health...you have to have self-funding programs...There’s no way you can...pull it from the bottom line because hospitals are already running as efficiently as possible...there is no fat and so, you need to make every program you do self-sufficient.

SH05; rural hospital; interview

Future sustainability of Patient Watch requires leveraging existing funding structures and avoiding reliance on short-term funding options:

...part of that was how this was always going to be funded and how we could tweak the funding to try and meet what we were doing...

SH10; lead agency; interview

[Patient] Watch is a, for every contact we’re actually funded by the government, so we’re got a different type of funding. So, we’ve got a continual stream.

SH01; lead agency; interview

Access to existing funding was initially used to support health coach training and activity and subsequently the training of and activity generated by TNS workers. However, 9 months into the spread, additional funding was leveraged to independently support TNS workers. Participants reported existing funding structures were “10-20 years old” (SH10; lead agency; interview), predating the recent shift toward virtual care, and suggested existing funding structures may change with changes in health care delivery. Furthermore, funding to maintain the program was seen as an important income stream to support patients, the hospital, and the broader community:

I think it’s still from an income stream, from a hospital perspective, you know, it’s the more contacts we get, the more money we generate, the, you know, the more we can support the region and our community.

SH01; manager; lead agency; interview

Funding was also perceived to be complex due to the different funding models hospitals were eligible for based on the delivery of health care across the rural region. The lead agency was activity based in contrast to the smaller rural hospitals that were block funded. Funding model differences meant that transfer of funds between health care services (even for the health care services that were part of the amalgamation) required a complicated solution, subcontracting to block-funded sites to receive payment from the activity-funded lead agency. Issues with subcontracting resulted in at least one health service being unable to implement Patient Watch. Furthermore, upfront investment was required for the initial implementation of Patient Watch (eg, training of health coaches) at all sites without patient activity funding to cover costs. While subcontracting addressed administrative process constraints, participants suggested the arrangement was inflexible and insufficient for supporting initial health coach training or complex patients, particularly in those areas without a local HARP team:

I spoke to one of my colleagues at another service who’s had [Patient Watch] running for at least six months and they’ve got about 40 clients that they’re looking after, the clients love it, the hospital board loves it, but the budget hates it because they’ve got such a dent in their budget because they can't pay for it.

SH05; executive; rural hospital; interview

##### System Integration

Participants highlighted the importance of system integration, that is, linking IT systems for the spread and scale-up of Patient Watch. There was a perception that system integration was difficult because of the diverse and outdated systems, the lack of funding for integration, and multiple third parties supporting system needs across the rural region. Health service amalgamation compounded system integration challenges because the priority was to operationalize systems for the amalgamation, while integration to support the spread and scale-up of Patient Watch was of lower priority. Similarly, the rural health alliance responsible for digital transformation and support of new patient programs was working with their own “antiquated programs that are very difficult and clunky” (SH05; rural hospital; interview) and were unable to deliver system integration across the rural region. Although the lack of system integration unintentionally created silos (eg, restricted information sharing) within health services and across the rural region, the spread and scale-up of Patient Watch was enabled by an agile workforce responding to the challenges through proactive, adaptable, and flexible work practices:

I would say that IT has probably been the issue that’s held us up for quite some time before we could move. I think that’s quite difficult and because you know we’ve all merged under [health service]. Even though we’ve merged not all the systems have merged, so there are still blocks and silos and things that happen that you have to try and get around or make a shortcut to get around or think outside the box, how can we do that?

SH02; lead agency; interview

However, solutions were often complex, manual, time consuming, and inefficient. Participants were frustrated with the lack of regional system integration and the lengthy delays experienced seeking system support (eg, development of local algorithms for case finding and reports for audit and feedback purposes). Furthermore, participants perceived that IT was underresourced and suggested a need for governance structures with a shared understanding of the roles and responsibilities for IT and the steering committee to better support the spread and scale-up of Patient Watch. Difficulty with system integration remained an ongoing challenge for spread and scale-up throughout the 20-month period of the study.

##### Workforce Capacity

The scale-up of Patient Watch was impacted by the uneven distribution of health care services and the health workforce characteristic of a rural context. Some areas within the rural region lacked a sufficient workforce (eg, could only support a part-time health coach; rural hospitals 1 and 4) or were not able to find a suitably qualified candidate for the role (rural hospital 3). For these areas, the health coach role was assigned to the closest available health coach. Other areas recruited health coaches with a range of different clinical backgrounds (rural hospitals 1 and 4; eg, enrolled nurses and social workers), acknowledging workforce challenges had driven the shift, with participants suggesting “job creep” (SH05; rural hospital; steering committee observation) was common and saying “we have to take what we can get” (SH05; rural hospital; steering committee observation).

The uneven distribution of health care services and the health workforce across the rural region delayed access to preventive and primary health care for patients because a key component of Patient Watch was the coordination of care with appropriate community-based health and social services. Spread and scale-up across the rural region was difficult because the ability to coordinate care was impacted by the perceived GP shortages that meant patients often had long wait times for appointments, less access to GPs due to the shift away from bulk billing services, and limited access to after-hours care:

Access to regular doctor’s appointments. I mean, there’s just, the doctor service is lacking. I think it, as it is in most places, getting in to see a doctor. Also, the introduction of fees, because there used to be a clinic in [rural area] that offered bulk billing, and that’s no longer, they’re now charging.

SH09; rural hospital; interview

Similarly, participants suggested there were inadequate mental health services, with some areas in the rural region more severely impacted (rural hospitals 1, 3, and 4). Participants from some areas also said that some services were not available (eg, HARP; rural hospitals 1, 4, and 5) and highlighted shortages in publicly funded allied health services, care services, and other resources (eg, diabetes educator; rural hospital 3). For areas without services, the default practice was referral to the large regional center, requiring patients to travel long distances with limited public transport options:

So, rurally, all of my clients that have mental health issues have to go to [the Regional Centre]. Well, that’s hard if you’re in [a small rural town] and you have no transport...

SH05; executive; rural hospital; interview

#### Theme 2: Context Drives Adaptation and Innovation

##### Overview

This theme represented the importance of adaptation and captured innovation that occurred in response to the context. The factors identified in theme 1 (funding, system integration, and workforce capacity) had important implications for adaptation.

##### Adaptation

Adaptation was a critical strategy underpinning spread and scale-up, with Patient Watch tailored to each health service to meet local needs. Adaptation occurred throughout the spread and scale-up, with adaptations to core components of the metropolitan model in response to challenges unique to the rural region. Participants reported considerable geographic distances, and the dispersed population across the rural region as the key drivers for adaptation. Within 2 months (early spread) of enrollment of the first patients by the lead agency, Patient Watch was adapted to provide virtual care (telehealth) using generalist health coaches (registered nurse) and without an allied health team in comparison to the metropolitan model providing home-based care using specialist health coaches and an in-house allied health team:

So, we’ve actually changed from being a fully visit the person in the home for their initial assessment to working within the office and doing phone calls and telehealth. So, wasn’t overly viable in health coach time and financially for us to be doing home visits.

SH02; lead agency; interview

Scale-up across the rural region progressed with the adapted virtual care model. Smaller rural health services made additional adaptations throughout scale-up regarding Patient Watch delivery and the target population, for example, trialing a direct referral model from local GPs or community health centers to enable a sufficient case load and funding for local health coach capacity (rural hospital 1), expanding the role of TNS workers to mitigate high staff turnover because of the adverse effects associated with calling patients in distress (rural hospital 1), and a focusing on appropriate referrals to community services rather than provision of health literacy (rural hospital 1) compared to the lead agency.

Adaptation of existing health system processes was also required to achieve alignment and involved workflow redesign (eg, enrollment of patients from the smaller rural hospitals [rural hospitals 2-5] to the lead health agency as required by the subcontracting arrangement) and remained in place throughout scale-up. The spread and scale-up of Patient Watch was supported by new software supporting clinical information management and required adaptation of existing processes to align software platforms within and across health services:

There’s still problematics between [the digital medical record and the new software], that there’s no automatic connection, so we have to manually upload documents...like the consultation notes of the nurse and the periodic reviews of the nurse into [the digital medical record] manually.

SH02; lead agency; interview

Adaptation was facilitated by the flexibility to tailor the program to patient need (eg, adjust duration and dose of Patient Watch), flexibility in the scope of the TNS worker and health coach roles, and flexibility in implementation processes to align with local health service needs. Adaptation was often reactive and in response to opportunities, challenges, and changes. Adaptation was a continuous process, fostering increased innovation and responsiveness as the context changed over time. The agility to pivot the program, strategies, and processes in response to the changing context was a key factor supporting the spread and scale-up of Patient Watch:

It evolves every couple of weeks to adapt to what we need and the services we need to deliver. I think it’s important to keep your eyes open to growth, rather, and change rather than keeping it as a closed caption idea.

SH02; lead agency; interview

##### Compatibility and Ease of Integration

Responsibility for the spread of Patient Watch at the lead agency lay within the directorate responsible for HARP, a service providing support and care for people with chronic and complex conditions to reduce avoidable hospital admissions and ED presentations. Strong engagement of the key stakeholders enabled alignment and integration of the 2 programs. For example, initial training of health coaches and TNS workers was provided through the HARP program, and the metropolitan Patient Watch SOPs were adapted to existing HARP processes using HARP resources, such as best practice guidelines:

...it felt really natural, I guess, to use all of those resources, skills, knowledge and experience to, to get the staff up and going, for [Patient] Watch, and, yeah, and then work between the two, like to try and find the best place for the patient.

SH06; lead agency; interview

One of the most exciting innovative developments was the development of a shared referral pathway to transfer patients between the 2 programs and occurred before the induction of the first patients to Patient Watch (before spread). Patients with chronic and complex conditions requiring multidisciplinary care were “stepped up” to HARP, and patients with less intensive needs were “stepped down” to Patient Watch:

If the patient was really complicated because [Patient] Watch doesn’t have allied health associated, we’ve only got nurses and the TNS team...then we would move them across to HARP. So, when HARP has them for a long time and they’re ready for discharge, rather than just having high care given in HARP and then just going, you’re off on your own, they’re actually stepping them across to us [Patient Watch] so that they feel a little bit more supported for a few weeks before they’re stepping out of services.

SH02; lead agency; interview

This mutually beneficial arrangement allowed Patient Watch access to intensive home-based multidisciplinary care and HARP to manage waitlists by supporting patients with an extended length of stay, acting as a bridging service while waiting for HARP services, and creating access to patients falling through referral gaps:

...you have to refer into HARP. You can self-refer, a health professional can refer, family member can refer, but we rely on a referral. And I think that was the biggest gap is that all these people are in ED today, we may not get a referral for any of them. Any of them. And I think that’s why I sort of thought [Patient Watch], that might be that gap filler because you didn’t have to rely on referral.

SH06; lead agency; interview

The development of the shared referral pathway was unique to the lead agency (ie, not part of the metropolitan model), and its success meant this was intended to be scaled across the rural region where possible (rural hospitals 2 and 3). Notably, not all rural hospitals had a local HARP team (rural hospitals 1, 4, and 5). Furthermore, the success in the alignment of the 2 programs resulted in the inclusion of ED care coordinators to align the 3 programs together at the lead agency.

#### Theme 3: Autonomy

This theme referred to the extent to which decision-making power was centralized and the degree of autonomy across the rural region. It recognized that centralized decision-making impacts administrative processes, access to funding (described in theme 1), capacity building in the rural region, and future sustainability of Patient Watch.

The advantages of a centralized approach included the lead agency providing administrative support and health coach training and resources (eg, SOPs and workflow plans) to the smaller rural health services. Furthermore, health coaches from the lead agency supported services unable to find local health coaches or provide cover 5 days per week and also escalations in care. Although the lead agency played a critical role in enabling the move from conception to spread and scale-up, there were differing visions across the region for the current program and for the potential of the future program.

Rural sites had some negative perceptions of the centralized approach related to the perceived narrow vision to address frequent presentations to the ED, the lack of focus on smaller rural health services, and the limited ability to innovate locally.

I think one of the other, the barriers probably are...it’s a real focus on ED...but there really isn’t a focus on, on rural and how that’s working rurally and it doesn’t feel like, umm, that we actually pay enough attention to that...the common factors are about what we’re doing at [lead agency]...whereas the client who lives on the farm at the back of [rural area] is just as important.

SH05; rural hospital; interview

During spread at the lead agency and early scale-up for rural sites, participants perceived an unwillingness to deviate from the central plan and less focus on rural needs and patient differences (eg, patient cohorts differed across the rural region with some cohorts considered more complex, HARP or mental health services that normally supported these patients did not exist, or insufficient numbers identified from the algorithm to support a local health coach). As noted by several participants, there were substantial differences across the rural region, requiring bespoke solutions to different challenges:

I’ve actually done a trip around the region recently and the challenges are different everywhere. It’s quite extraordinary actually.

SH06; lead agency; interview

...unlike what we’ve got in [metropolitan areas] or even in [site of lead agency], these clients live, some of them were living off grid, some of them live in shacks, some of them live in, so no power, no water. So, there’s some really different, and there’s miles out in the bush. So, there’s a whole different process in regards to that...

SH05; rural hospital; interview

There was a strong consensus that health coaches should live and work in the community they served because a deep understanding of the community and its resources was necessary to undertake the role effectively:

When the program was being set up, they started setting up that [health coaches] could all be here in [lead agency] and most of the regions wanted a local health coach for the local region because they knew their services...They live it every day, so it is a reasonable request to have it in their area.

SH02; lead agency; interview

Furthermore, the establishment of relevant partnerships was location dependent, with local relationships considered important for scale-up to engage in continuous problem-solving and adaptation. For example, the development of the step-up or step-down process between Patient Watch and HARP at the lead agency was bespoke (eg, rural hospitals 1, 4, and 5 did not have HARP) and highlighted the need to develop relevant local partnerships. In addition, engagement with HARP at rural hospitals 2 and 3 was independent of the lead agency and occurred well after scale-up had already started in these areas (early scale-up to mid–scale-up).

## Discussion

### Principal Findings

This study addressed a significant gap in knowledge of the spread and scale-up of an effective telehealth case management intervention from a metropolitan to rural context. Our study provides evidence that the spread and scale-up of Patient Watch was dynamic and nonlinear and required ongoing model flexibility to respond to local contexts. One potential advantage of telehealth programs such as Patient Watch is the ability to deliver services remotely from a centralized location, resulting in cost savings and access benefits, which has much appeal to rural areas. However, the numerous challenges that arose with the implementation of Patient Watch seem widely applicable in telehealth scale-up to rural areas. The complexity of the health system meant preexisting system factors (eg, financial resources, workforce constraints, and infrastructure challenges) influenced organizational capacity for spread, scale-up, and future sustainability. Our findings also suggest that continuous contextual adaptation in response to system factors was essential for tailoring to better meet local organizations’ needs and capacities in a rural context. Autonomy for smaller rural hospitals helped to facilitate adaptation to address variations across local contexts, while central governance restricted tailoring of Patient Watch to local contexts.

The dichotomy of the rural-metropolitan divide has informed conceptualizations of rural and metropolitan areas and flattened the complexity of rural areas by ignoring the diversity of demographic, social, economic, and health system characteristics [31,45]. Our study emphasized the importance of adapting Patient Watch to a rural context and in response to the nuances and diversity of rural contexts. We challenge the “deficit” perspective of rural health systems and contribute to shifting the paradigm by recognizing their strengths. Despite few interventions being developed or adapted for rural areas and in response to the diversity of rural areas [22,23], Patient Watch achieved spread and scale-up. However, with such considerable adaptation to the program, it warrants the consumer impact to be re-established. Key strengths were the region-wide collaboration among rural health services; identification of resources, including leveraging of funds; and tailoring of Patient Watch to respond to the local context by an agile, flexible, and adaptable workforce. These strengths highlight the value of working at a regional level to coordinate efforts and share resources for delivering a high-performance health system, improving the health of rural populations.

Our study suggests the need to consider the complexity of the system in which telehealth case management interventions are embedded and the influence of the external context on organizational capacity for spread, scale-up, and future sustainability. Key contextual factors included the availability and access to funding, the lack of system integration and interoperability, and challenges with rural workforce capacity. Funding, whether as a national, state, or local strategy and sustained funding through government organizations, has been identified as critical for implementation [46,47]. Although evidence for the influence of system integration on spread, scale-up, and sustainability is lacking, some evidence suggests that fragmentation of connections among hospitals resulted in duplication of work and less efficiency compared to hospitals with seamless connections [48]. Evidence also suggests that longstanding rural health workforce shortages and high turnover substantially impact the successful implementation of interventions in a rural context [22]. These findings align with the NASSS framework, which highlights the dynamic and interrelated factors that influence the adoption and outcomes of health technology interventions [30]. Particularly, the wider institutional and sociocultural context in this framework was considered pivotal for moving from a successful demonstration project to spread, scale-up, and sustainability [30]. Generally, there is a lack of evidence focusing on the influence of the external context, and it is often not the focus of implementation research [46,47], possibly because it is perceived as more difficult to change [47]. We suggest that in any given context, geographic, financial, political, technological, and sociocultural influences interact and interconnect, resulting in complex and unpredictable outcomes. This means that there is no simple or standardized way of implementing many complex interventions, including telehealth case management, at scale in a complex system; we recognize that the spread and scale-up efforts grow organically within their context, highlighting the importance of place-based approaches and co-designing for success.

Our study also demonstrated why many interventions cannot simply be transferred across settings as a self-sufficient, “generic” package and supports recent views that establishing an intervention represents only the beginning of model optimization. The core components, mode of delivery, service setting, and target audience of Patient Watch required significant adaptation to facilitate spread, scale-up, and future sustainability, with adaptations primarily driven by the pressure to enact government policy quickly and the need for financial stability. In a real-world rural context, the practical application of implementing at scale was a process of “learning on the fly” and adapting accordingly and differs from a traditional linear research approach. There is increasing recognition that adaptation is essential in real-world contexts [29], including in rural settings implementing interventions developed in a metropolitan setting [22,23,31]. Our results align with DSF, which emphasizes constant change in a multilevel context, the intervention itself, the context in which the intervention is delivered (eg, organizations), and the broader health system in which the organization operates [29]. The DSF also suggests that continuous and iterative adaptation is not just an unfortunate or unavoidable consequence of implementation; it is critical for sustainability and continued health service fit within a changing context [29]. Furthermore, Côté-Boileau et al [26] recognized the unpredictability of context and its influence on spread, scale-up, and sustainability and suggested that continuous contextual adaptation is essential to bring alignment between the innovation and organization needs and capacities. Similarly, we suggest that adaptation is central to spread, scale-up, and sustainability, and adaptive capability is essential at the system, organization, and intervention level.

The spread and scale-up of the telehealth case management intervention was not linear, predictable, or standardizable and must allow for local tailoring to address barriers and increase fit with contextual factors. Our findings suggest that by having autonomy, organizations can self-organize (ie, the ability for local staff to initiate adaptations to complete tasks given locally available resources and contexts) and help address variations across local contexts. Self-organization has been suggested to be a powerful strategy for improving the spread, scale-up, and sustainability of interventions [49]. However, centralized governance and decision-making have been shown to be in tension with self-organization. While centralized governance provided advantages (eg, capacity building and administrative support), there were also disadvantages (eg, a lack of local autonomy restricting tailoring of Patient Watch). It has been suggested that decentralized governance supports increasing autonomy of local managers and fosters innovation, while centralized organizational structures stifle innovation [50,51]. Distributed leadership within and across interprofessional and interorganizational boundaries has been suggested to increase capacity building needed to support and operationalize spread, scale-up, and sustainability [26]. In contrast, structural hierarchies and accountability appear to hinder spread, scale-up, and sustainability [26]. Responding to the local context is important to enable complex health systems and organizations to increase uptake of telehealth case management interventions by ensuring a better fit with local needs and capacity. Rosenberg et al [52] reflected on mental health planning in Australia and described centralized governance as unable to overcome a complex system, resulting in a “complexity gap” and making decisions without understanding the local context. Deliberately leveraging self-organization may be a powerful strategy to achieve implementation at scale across diverse contexts; however, achieving the right balance between decentralized and centralized governance warrants further exploration.

### Strengths and Limitations

A strength of this study was the qualitative approach to analyze and explore complexity and gain a deeper understanding of rural contexts. The engagement of a range of stakeholders from frontline staff to decision-makers was key to capturing a range of perspectives and experiences. Attendance at the steering committee meetings and document analysis increased participation of a broader group of stakeholders and enhanced the understanding of findings. We acknowledge several limitations. The semistructured interviews were conducted 18 months after the implementation started and may have missed capturing early or late implementation efforts. Perspectives and experiences may change as learnings and knowledge from early implementation efforts are likely to influence ongoing implementation efforts. Greater insights may be gained by interviewing a more diverse representation from rural areas and including patient participants.

### Conclusions

In conclusion, the findings emphasized the key factors needed to facilitate the implementation of a metropolitan-centric program at scale in a rural context. We suggest that health system factors influence organizational capacity, adaptations are essential to address rural contextual issues, and a balance of a central approach through larger sites and self-organization is needed. Our findings also challenge the “deficit” perspective of rural health and highlight the strengths of a rural context to efficiently achieve spread, scale-up, and sustainability. Future research should embrace health system complexity, build on comprehensive resources, commit to continuous adaptations in different contexts, and integrate a balance of distributed leadership to enhance the spread, scale-up, and sustainability of telehealth case management interventions.

## Appendix

Multimedia Appendix 1 Semistructured interview schedule.

## Abbreviations

DSF: dynamic sustainability framework
ED: emergency department
GP: general practitioner
HARP: Hospital Admissions Risk Program
MM: modified Monash
NASSS: nonadoption, abandonment, scale-up, spread, and sustainability
SOP: standard operating procedure
TNS: tele-navigator support
