# Supplementary material for: Spread and Scale-Up of a Region-Wide Telehealth Navigation Service in a Rural Context: Qualitative Process Evaluation
Source: J Med Internet Res. 2025 Jun 23;27:e64734. doi: 10.2196/64734 (PMC12235203; doi:10.2196/64734)
Supplement: Multimedia Appendix 1 [file jmir_v27i1e64734_app1.docx]

### Multimedia Appendix 1: Semi-Structured Interview Schedule

1. How was Patient Watch chosen as the program to be implemented?
2. Were other programs considered?
3. What was the intention of the program starting out and what do you see as the purpose of the program now?
4. What are the anticipated outcomes?
5. What was the driving need for Patient Watch in site 1 and across the region? What were you seeking to address? What was the State Government seeking you to address?
6. Was the original intention to roll out Patient Watch to the regions? How has the amalgamation of the health services influenced the intention to roll out Patient Watch to the regions?
7. What kind of adaptations have been attempted to make Patient Watch work in site 1 and across the region?
8. Where did the funding come from to support implementation of Patient Watch?
9. How has the funding been used to support imple¬¬mentation in site 1 and across the region?
10. Is there additional funding that can be accessed to support ongoing implementation in site 1 and across the region? If so, what funding is available?
11. How important is it for Patient Watch to be self-supporting in site 1 and across the region?
12. Have there been any unexpected costs or funding constraints associated with the implementation of Patient Watch in site 1 and across the region?
13. How well suited is Patient Watch to being rolled-out across the region?
14. How has the infrastructure of main health service in site 1 and across the region influenced the implementation of Patient Watch?
15. How does the program fit with existing work processes and practices in site 1 and the regions e.g., integration with other programs, priority to implement, competing priorities?
16. What have been the barriers to implementing Patient Watch in site 1 and across the regions? To what extent are these barriers common to the roll out of other programs?
17. What have been the enablers to implementing Patient Watch in site 1 and across the region?
18. Who needs to do what to make Patient Watch a success in site 1 and across the region?
19. How can Patient Watch be sustained over time?
20. What will happen over the long-term to Patient Watch?
21. Are there plans to leave the program in place in site 1 and across the region??
22. What are the measures of success required to ensure Patient Watch remains embedded long-term in site 1 and across the region?
23. Are there plans to expand the program in site 1 and across the region e.g., mental health stream?
